# CD4^+^ regulatory and naïve T cells specific for factor VIII stand vis‐à‐vis to balance the immune response in healthy individuals

**DOI:** 10.1002/jha2.319

**Published:** 2021-11-02

**Authors:** Anja Schmidt, Aleksander Orlowski, Emilia Salzmann‐Manrique, Christoph Königs

**Affiliations:** ^1^ Department of Paediatrics and Adolescent Medicine, Clinical and Molecular Haemostasis University Hospital Frankfurt, Goethe University Frankfurt am Main Germany; ^2^ Department of Paediatrics and Adolescent Medicine, Division for Stem Cell Transplantation and Immunology University Hospital Frankfurt, Goethe University Frankfurt am Main Germany

Haemophilia A (HA) is an inherent coagulation disorder with a reduced or absent factor VIII (FVIII) activity and is usually treated by FVIII replacement therapy. About 30% of previously untreated patients with severe HA develop inhibitory antibodies towards FVIII rendering replacement therapy ineffective [1]. Additionally, inhibitory antibodies to FVIII rarely occur in healthy individuals resulting in acquired HA. Thus, very few healthy individuals (0.2–1.0 per one million) [2], as well as one‐third of HA patients, mount an aggressive auto‐ or alloimmune response to FVIII. FVIII‐specific immune responses are usually analysed and quantified on a FVIII‐specific humoral level. However, specific CD4^+^ T cells need to be present as well to help induce this humoral immune response. Of note, several studies report the existence of FVIII‐specific CD4^+^ T cells in healthy individuals [3, 4].

Surface markers for early detection of antigen‐specific T cells following antigen‐stimulation have been identified some years ago [5]. CD154 (CD40L) identifies non‐regulatory activated T cells, while CD137 (4‐1BB) is the corresponding surface marker for activated regulatory T cells (Tregs). This approach has recently also been used to identify SARS‐CoV‐2‐specific T cells [6]. Here we used this approach to analyse FVIII‐specific immune responses in healthy individuals.

For this, we isolated peripheral blood mononuclear cells (PBMCs) from 19 buffy coats (Blutspendedienst, Frankfurt am Main, Germany) of healthy individuals by density gradient centrifugation using Histopaque‐1077 (Sigma Aldrich, San Louis, MO, USA). Each 1×10^6^ PBMCs were resuspended in 100 μl RPMI with 1% penicillin/streptomycin, 2% L‐glutamine, and 5% hAB serum (all Sigma Aldrich), containing 1 μg/ml anti‐CD28 antibody (clone CD28.2; Biolegend, San Diego, CA, USA) and 1 μg/ml anti‐CD40 antibody (Miltenyi Biotec, Bergisch Gladbach, Germany). Cells were stimulated for 16 h with different concentrations of recombinant, full‐length FVIII (rFVIII), the fusion protein rFVIIIFc and myelin‐oligodendrocyte protein (MOG) as an autoantigen control. Unstimulated cells served as negative, cells stimulated with 5 μg/ml anti‐CD3 antibody (clone OKT3; Biolegend) as positive control.

Following stimulation, cells were analysed by flow cytometry using antibodies against CD8, CD56, CD19, CD14, CD25, CD45RA, CD154, CD137, CD127, CD197 and CD4 (all Miltenyi Biotec, see Table S1 for conjugates and clones). Cells were classified into Tregs and non‐Tregs based on CD25 and CD127 surface expression and further subdivided into naïve, effector and memory cells using markers CD45RA and CD197. Antigen‐specific activation was determined by surface expression of CD137 for Tregs and CD154 for non‐Tregs (see Figure 1 for gating strategy). The endpoints of the study were the responsiveness to stimuli rFVIII, rFVIIIc and MOG with different antigen concentrations (1 IU/ml, 1 IU/100 μl and for MOG 740 pM and 7.4 nM) as rate and intensity. Statistical analyses were performed using the statistical software R, version 4.03 (R Project for statistical computing) and GraphPad Prism version 6.0.

**FIGURE 1:**
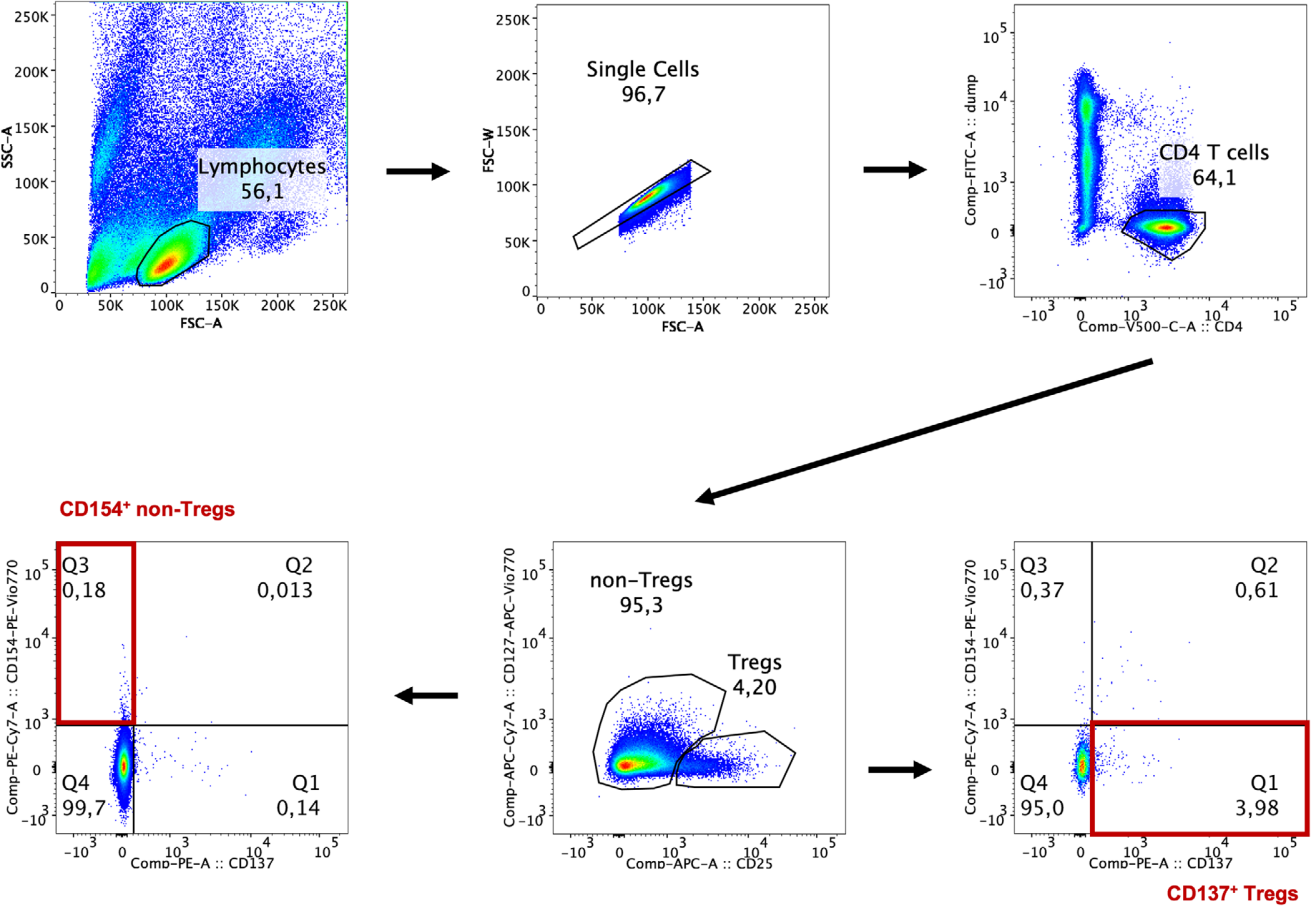
Gating strategy for identification of antigen‐specific CD4^+^ regulatory and non‐regulatory T cells. Following the exclusion of doublets and dead cells, CD4^+^ T cells were separated into regulatory T cells (Tregs) and non‐Tregs based on the expression of CD25 and CD127. Only CD154^+^ CD137^–^ non‐Tregs and CD154^–^ CD137^+^ Tregs were considered to be antigen‐specific.

Antigen‐specific responses were expressed as a ratio of antigen‐stimulated and non‐stimulated CD137^+^CD154^−^ Tregs or CD154^+^CD137^−^ non‐Tregs. Rates of responsiveness were compared using Fisher's exact test. CD25^+^CD127^−^ Tregs and remaining CD4^+^ non‐Tregs of all 19 samples were activated by the anti‐CD3 positive control. The portion of samples reacting to any of the other stimuli varied between seven and 12 out of 19 samples for non‐Tregs, and between five and 16 out of 19 samples for Tregs. A summary of the results is presented in Table S2. As a positive correlation was found between the antigen concentrations and their specific response rates, further results will focus on samples with higher concentrations used only. While differences in the fraction of reacting CD154^+^ non‐Tregs for the different stimuli were not significant, Tregs reacted significantly more towards 1 IU/100 μl rFVIII (84.2%) and 1 IU/100μl rFVIIIFc (68.4%) compared to MOG (26.3%) (*p *< 0.001), as observed by the expression of activation marker CD137 (Figure 2A). Additionally, the distributions of intensities between groups were compared using Wilcoxon signed‐rank test or Friedman Test as appropriate. Here, Tregs showed a higher intensity of responsiveness against rFVIII and rFVIIIFc than against MOG with a median ratio of 1.39 compared to 1.11 (rFVIIIFc) and 0.88 (MOG) (*p *< 0.001) (Figure 2B and Table S3).

**FIGURE 2:**
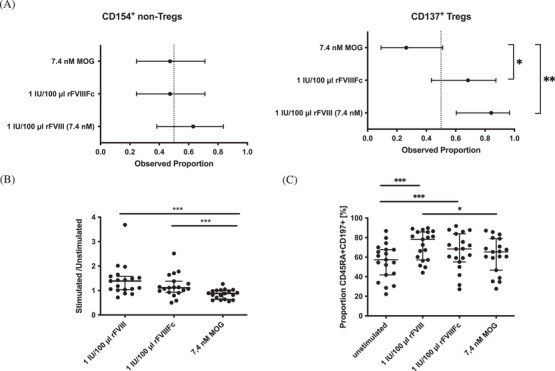
Response of activated regulatory T cells (Tregs) and non‐Tregs towards factor VIII (FVIII) and myelin‐oligodendrocyte protein (MOG). Following stimulation of peripheral blood mononuclear cells (PBMCs) isolated from buffy coats of healthy individuals with full‐length FVIII (rFVIII), the fusion protein (rFVIIIFc) and MOG for 16 h, the proportion of samples responding to the stimuli based on surface expression of CD154 (non‐Treg) or CD137 (Treg) was compared. MOG was used in an equivalent molar concentration as rFVIII. A total of 85,421 to 125,377 (median: 97,982) CD4^+^ T cells was acquired. (A) The rate of responsiveness to stimuli is presented with a two‐sided 95% Clopper‐Pearson confidence interval (CI95). Intensities of responsiveness were compared using Wilcoxon signed‐rank test or Friedman Test. Tregs reacted more often against rFVIII and rFVIIIFc than against MOG. (B) When comparing the intensity of the response of stimulated Tregs, the intensity was higher for the response towards rFVIII and rFVIIIFc. (C) CD154^+^ CD137^–^ non‐Tregs were further classified based on markers CD45RA and CD197. The proportion of naïve CD45RA^+^ CD197^+^ non‐Tregs reacting to the stimuli was compared and was higher for rFVIII and rFVIIIFc compared to the unstimulated control. Of interest, the proportion of naïve cells responding to rFVIII was also significantly higher than the proportion of MOG‐specific naïve cells. All tests were two‐sided and a *p* < 0.05 was considered significant.

While no significant differences were found for the responsiveness of the whole CD4^+^ non‐Treg population towards the antigen‐specific stimuli used in this study, the proportion of CD45RA^+^CD197^+^ naïve T cells expressing activation marker CD154 following in vitro incubation was highest for rFVIII‐stimulated cells (median of 78.2%) and statistically different to MOG (median of 65.4%) (*p *< 0.05) (Figure 2C and Figure S4). Both, rFVIII and rFVIIIFc stimulation (median of 68.4%) showed a higher proportion of activated naïve T cells compared to the unstimulated control (median of 57.4%) (*p *< 0.001 and *p *= 0.002). Thus, in this setting naïve CD4^+^ T cells showed higher responsiveness towards either rFVIII product.

Of note, PBMC samples analysed were collected from seven (37%) women and 12 (63%) men, aged 19–61 years, the median age being 36 years. There was no significant age difference between male (median 48; range 21–61) and female (median 32; range 19–43) volunteers (*p* = 0.128). In addition, no association was detected between sex or age and the rate or the intensity of the responsiveness.

Interestingly we were able to detect FVIII‐specific T cells in our study although only a median of 97,982 CD4^+^ T cells was analysed after stimulation of 1 × 10^6^ PBMCs. An earlier study focussing on FVIII‐specific T cells in healthy individuals assumed that 2–4 out of 1 × 10^6^ CD4^+^ T cells in this cohort are FVIII‐specific [3]. Our results suggest that the number of FVIII‐specific cells is higher than previously expected. Alternatively, stimulation with FVIII might lead to a higher amount of activated bystander T cells. However, from a clinically relevant point of view, we think that every T cell activated by FVIII, either specifically or as a bystander, would have an impact on the FVIII‐specific immune response in a FVIII‐treated individual.

Our results point out, that the naïve CD4^+^ non‐Treg repertoire appears to have a higher quantity of rFVIII‐responsive cells compared to the MOG autoantigen control. Additionally, while only five out of 19 donors had MOG‐specific Tregs, 16 out of 19 donors had FVIII‐specific Tregs. To our knowledge, this study is the first one reporting the existence of FVIII‐specific Tregs in humans.

While the proportion of responding naïve T cells was highest for rFVIII, the difference between the responses towards rFVIII and rFVIIIFc was not significant. Interestingly, no difference in total response and response intensity was detected for the whole CD4^+^ non‐Tregs while Tregs reacted more often and stronger towards rFVIII and rFVIIIFc compared to MOG. Different mechanisms of tolerance against auto‐antigens have been described. While reactive T cells during thymic development can be induced to undergo apoptosis, they can also be reprogrammed to become anergic or regulatory T cells [7]. Though, the development of tolerance against self‐antigens certainly is not absolute. Concerning reactivity towards FVIII, our data suggest that a significant proportion of healthy individuals possesses FVIII‐specific T cells and that specific Tregs are produced during early tolerance development to bridle this cell population. Thus, situations in which Treg functions are impaired might result in the formation of robust FVIII‐specific T‐cell responses eventually leading to the formation of FVIII‐specific antibodies. The existence of FVIII‐specific antibodies in healthy individuals [8] supports the hypothesis of a not perfectly balanced T‐cell response towards FVIII in these individuals. Proving the regulatory function of CD137^+^ Tregs would substantially support our hypothesis, that FVIII‐specific Tregs present in healthy individuals are usually able to suppress FVIII‐specific immune responses. However, due to the low cell counts of FVIII‐specific cells in the original samples, functional suppression assays would only be possible following several rounds of T‐cell expansion. We are currently developing protocols for further functional analysis of FVIII‐specific T cells within PBMC preparations.

This study adds a novel aspect to understanding the allo/autoimmune response to FVIII. Of note, existing FVIII‐specific CD4^+^ T cells might still be anergic and therefore hampered in inducing an FVIII‐specific immune response even in the absence of FVIII‐specific Tregs. A detailed characterisation of the responsive cells should be addressed in future experiments.

In order to better understand differences between the response of PBMCs from healthy individuals towards rFVIII or other auto‐antigens, additional data about the secretion of cytokines by T cells but also B cells and monocytes are needed. Of course, the analysis of FVIII‐specific Tregs and also other immune cell populations in HA patients, also in relation to the inhibitor status, will be extraordinarily important to potentially identify predictive cellular markers for the formation of FVIII inhibitors in HA patients. This study identified FVIII‐specific Tregs in healthy individuals and adds to the understanding of the immune response to FVIII. There are limitations when using these data to interpret the immune response to FVIII in HA patients. However, the analysis of healthy individuals could help to identify specific characteristics of antigen‐related immune responses and in terms of FVIII also enables the identification of markers correlating with an increased risk for the development of acquired HA.

## Supporting information



supporting information

## Data Availability

Data are available on request from the authors.
