# Supplementary material for: CD4+ regulatory and naïve T cells specific for factor VIII stand vis‐à‐vis to balance the immune response in healthy individuals
Source: EJHaem. 2021 Nov 2;2(4):805–8. doi: 10.1002/jha2.319 (PMC9175863; doi:10.1002/jha2.319)
Supplement: Supplementary file 1 — supporting information [file JHA2-2-805-s001.pdf]

Supplemental Table 1: *Antibodies used for staining of stimulated PBMCs. All antibodies were obtained from Miltenyi Biotec (Bergisch Gladbach, Germany), the fixable viability dye eFluor520 was obtained from Thermo Fisher (Waltham, USA).*

Antibody Specificity	Antibody Conjugate	Clone
CD8	FITC	REA734
CD56	FITC	REA196
CD19	FITC	REA675
CD14	FITC	REA599
Viability	eFlour520	
CD25	APC	REA570
CD45RA	PerCP-Vio700	REA562
CD154	PE-Vio770	5C8
CD137	PE	4B4-1
CD127	APC-Vio770	REA614
CD197	VioBlue	REA546
CD4	VioGreen	REA623

Supplemental Table 2: *Responsiveness of CD4⁺ non-Tregs (A) and Tregs (B) based on expression of CD154 and CD137. The observed rates of reacting samples are presented descriptively with counts, percentages and its two-sided 95% Clopper-Pearson confidence intervals (CIs). Higher expression of markers in stimulated samples in comparison to the unstimulated control is presented as "1", a lower expression as "0". PBMCs of all analysed Buffy Coats responded to the anti-CD3 stimulus as positive control.*

A)

CD154-positive non-Tregs

Sample	1 IU/ml rFVIII	1 IU/100 µl rFVIII	1 IU/ml rFVIIIFc	1 IU/100 µl rFVIIIFc	740 pM MOG	7.4 nM MOG	anti-CD3 (5 µg/ml)
1	1	0	1	0	0	0	1
2	1	1	1	0	0	1	1
3	1	0	0	0	0	1	1
4	1	0	1	0	1	1	1
5	0	1	0	1	0	1	1
6	0	1	0	1	0	1	1
7	1	1	1	1	0	0	1
8	0	1	0	0	0	0	1
9	0	1	0	1	1	0	1
10	1	1	0	0	1	0	1
11	0	1	0	1	0	0	1
12	0	0	0	0	1	0	1
13	0	0	0	1	0	1	1
14	0	1	0	0	1	1	1
15	0	1	1	1	1	1	1
16	1	1	1	1	1	0	1
17	0	1	1	1	0	0	1
18	0	0	0	0	0	1	1
19	0	0	0	0	0	0	1
**Total**	**7/19**	**12/19**	**7/19**	**9/19**	**7/19**	**9/19**	**19/19**
**Rate (%)**	36.8	63.2	36.8	47.4	36.8	47.4	
**95% CI**	(16.3-61.6)	(38.4-83.7)	(16.3-61.6)	(24.5-71.1)	(16.3-61.6)	(24.571.1)	

B)

CD137-positive Tregs

Sample	1 IU/ml rFVIII	1 IU/100 µl rFVIII	1 IU/ml rFVIIIFc	1 IU/100 µl rFVIIIFc	740 pM MOG	7.4 nM MOG	anti-CD3 (5 µg/ml)
1	1	1	1	0	0	0	1
2	0	0	0	0	0	0	1
3	0	1	0	0	0	0	1
4	1	1	1	1	1	0	1
5	0	0	0	1	0	0	1
6	1	1	1	1	1	1	1
7	1	1	1	1	1	0	1
8	0	1	0	0	0	0	1
9	1	1	0	1	1	1	1
10	0	1	0	1	0	0	1
11	1	1	1	1	1	1	1
12	0	1	0	1	1	0	1
13	1	1	1	1	1	0	1
14	1	1	1	1	0	1	1
15	1	1	0	1	0	0	1
16	1	1	1	1	1	1	1
17	1	1	0	1	0	0	1
18	1	1	0	0	0	0	1
19	0	0	0	0	0	0	1
**Total**	**12/19**	**16/19**	**8/19**	**13/19**	**8/19**	**5/19**	**19/19**
**Rate (%)**	63.2	84.2	42.1	68.4	42.1	26.3	
**95% CI**	(38.4-83.7)	(60.4-96.6)	(20.3-66.5)	(43.5-87.4)	(20.3-66.5)	(9.1-51.2)	

Supplemental Table 3*: Summary of medians, quartiles, and ranges for the intensity of responsiveness towards mentioned stimuli.*

	**1 IU/100 µl rFVIII**	**1 IU/100 µl rFVIII-Fc**	**7.4 nM MOG**
Median	1.39	1.11	0.88
1. Qu - 3. Qu	1.05 - 1.55	0.95 - 1.33	0.67 - 0.99
Min - Max	0.72 - 3.69	0.50 - 2.52	0.56 - 1.27

Supplemental Table 4: *Summary of medians, quartiles, and ranges for proportion of antigen-specific CD197^{+}CD45RA^{+} naïve non-Tregs reacting towards the mentioned stimuli.*

	**Unstimulated**	**1IU/100 µl rFVIII**	**µl rFVIIIc**	**7.4 nM MOG**	**anti-CD3**
Median	57.4	78.20	68.40	65.40	53.20
1. Qu - 3. Qu	42.60 - 67.65	58.70 - 85.65	57.10 - 79.75	53.00 - 74.90	42.00 - 61.40
Min - Max	22.2 - 86.8	44.10 - 89.90	27.20 - 91.90	27.70 - 87.10	28.70 - 79.30
